# Enhancement of Social Communication Behaviors in Young Children With Autism Affects Maternal Stress

**DOI:** 10.3389/fpsyt.2021.797148

**Published:** 2021-12-07

**Authors:** Dominik Laister, Giacomo Vivanti, Peter B. Marschik, Johannes Fellinger, Daniel Holzinger

**Affiliations:** ^1^Institut für Sinnes- und Sprachneurologie, Konventhospital Barmherzige Brüder Linz, Linz, Austria; ^2^Research Institute for Developmental Medicine, Johannes Kepler University Linz, Linz, Austria; ^3^AJ Drexel Autism Institute, Drexel University, Philadelphia, PA, United States; ^4^Olga Tennison Autism Research Centre, La Trobe University, Melbourne, VIC, Australia; ^5^Child and Adolescent Psychiatry and Psychotherapy, University Medical Center Göttingen and Leibniz Science Campus Primate Cognition, Göttingen, Germany; ^6^Centre of Neurodevelopmental Disorders (KIND), Centre for Psychiatry Research, Department of Women's and Children's Health, Karolinska Institutet, Stockholm, Sweden; ^7^Interdisciplinary Developmental Neuroscience, Division of Phoniatrics, Medical University of Graz, Graz, Austria; ^8^Institut für Linguistik, Universität Graz, Graz, Austria

**Keywords:** autism spectrum disorder, social communication behaviors, maternal stress, early childhood, Early Start Denver Model, intervention outcomes

## Abstract

Children with autism spectrum disorder (ASD) show difficulties in social communication behaviors, emotion regulation and daily living skills, and they frequently present with challenging behaviors. In parents of children with ASD, higher rates of stress and mental health problems have been reported than in parents of either typically developing children or children with other conditions. In this study, we tested whether maternal well-being changes with improved social communicative behaviors of children with ASD receiving early intervention. We examined developmental changes in 72 pre-schoolers and stress levels in their mothers (measured by the Parental Stress Inventory) before and after a 12-month community-based intervention program based on the Early Start Denver Model, a naturalistic developmental behavioral intervention targeting social communication. Multiple regression analyses showed that maternal child-related stress was predicted by changes in children's social communication behaviors (measured with the Pervasive Developmental Disorder Behavior Inventory). Gains in the early social communication behavior domain were the strongest predictor of post-intervention child-related maternal stress, surpassing adaptive behavior, language and non-verbal cognitive gains, and reduction in challenging behavior. These findings support the hypothesis that, in children with ASD, the acquisition of social communication behaviors contribute to improvements in maternal well-being.

## Introduction

Autism spectrum disorder (ASD) is characterized by difficulties in social communication, patterns of restricted/repetitive behaviors and interests, and onset in early childhood (1). Higher rates of chronic parental stress, reduced overall well-being, increased occurrence of anxiety and depression and lower parental self-efficacy have been reported for parents of children with ASD compared to parents of either typically developing children or children with other disabilities such as Down syndrome or cerebral palsy (2–6). Mothers of children with ASD often score higher on stress levels than fathers (7). These increased rates of parental stress suggest that ASD-specific core symptoms, such as social communication difficulties and restricted and repetitive behaviors, might contribute to diminished parental well-being (8). Additionally, comorbid conditions—such as attention deficit hyperactivity disorder (ADHD), oppositional defiant disorder (9), behavioral/emotional problems (10–12) and anxiety or depression (13, 14)—that are often comorbid with ASD might intensify maternal stress.

### Child Factors Impacting Parental Health

A number of characteristics and behaviors of children with ASD have been associated with parental distress, including autism severity (15, 16), social communication deficits (17, 18), and challenging behaviors (10, 17). Peculiar speech-language functions or atypical communication is often the parents' first and major concern that leads them to seek professional help (19). Especially difficulties in social communication continue—across the lifespan—to cause parental distress, including anxiety and confusion about their child's behavior and parental feelings of inefficacy (20). Furthermore, higher rates of aggressive and self-injurious behaviors have been reported in children with severe social communication deficits (21). The link between challenging behaviors and increased parental stress is well-established (22). In addition to communication problems and challenging behaviors, impairments in cognitive and adaptive functioning that frequently occur in ASD are major causal factors of parental stress (23, 24) and frustration (25).

### Effects of Intervention on Parental Well-Being

In his developmental systems model for early intervention Guralnick (26) described the bidirectional relationship between family factors (e.g., socioeconomic status, mental health, coping styles, and social support) and child development mediated by family-child interactions that include parent-child communication and socio-emotional connectedness. Stressed parents are less likely to engage in positive interactions with their children and to be actively involved in intervention programs. However, social and cognitive difficulties in affected children must be regarded as stressors that may impact parent-child interaction and may consequently increase parental stress. Intervention approaches that focus on child social communication behaviors are therefore expected to positively affect parental stress. Research into the effect of specific components of intervention programs targeting child behavior (e.g., communication, cognitive functioning, and adaptive skills) on parental stress remains limited. Ozturk et al. (27) reported that improvements in social communication skills following early intervention had a positive impact on maternal satisfaction and psychological distress as measured by the Depression Anxiety and Stress Scale (28). Additional research has shown that family-centered approaches that directly target family outcomes by providing socio-emotional support to families and/or parent training in positive interaction strategies with their child are associated with greater family involvement, self-efficacy and well-being (26, 29). Examples include the parent-implemented Early Start Denver Model (P-ESDM) intervention (10) and the PACT program (30), both of which have been associated with reductions in parental stress.

The aim of this study was to evaluate whether stress in mothers of children with ASD is impacted by changes in child behavior after a 1-year early-intervention program. To this end, we deployed a community-based intervention (31) utilizing the ESDM approach. The ESDM is an evidence-supported early-intervention program for children with ASD aged 1–4, with a focus on promoting social communication behaviors. The principles and strategies of ESDM are informed by behavioral and developmental research that highlights the importance of early social engagement and social communication in the development of children with ASD (32–37). Empirical support for the ESDM includes evidence of improvements in cognitive and communication functioning (38, 39).

Based on the body of knowledge gained through these studies, we hypothesized (1) that maternal child-related stress would decrease between baseline (T1) and post-intervention (T2) in mothers of children receiving ESDM intervention for 1 year; (2) that the enhancement of social communication behaviors between T1 and T2, assessed by parent report, predicts the outcome of maternal child-related stress at T2; and that (3) improvements in (a) verbal skills and (b) non-verbal cognition (both directly assessed with standardized instruments) and (c) adaptive behavior skills and (d) behavioral problems (both parent-reported) predict the outcome of maternal child-related stress at T2.

## Methods

The study was conducted at the Institute of Neurology of Senses and Language, hospital of St. John of God Linz, which provides multidisciplinary diagnostic and intervention services for children with developmental disorders, with a focus on communication disorders.

### Outcome Measures

#### Parenting Stress Index Short Form

The widely used Parenting Stress Index Short Form (40) is a self-report for measuring difficulties related to the parenting role. The questionnaire is available in German (41) and normed to German mothers of a non-clinical group of children and has good to excellent validity and reliability scores. The PSI-SF resulted from a factor analysis of the original long form with a correlation for the total stress scores of *r* = 0.94, *p* < 0.001 (42) and the subscales show high internal consistency, with alphas of 0.91–0.95 and test–retest reliability from 0.85 to 0.87 (41). The PSI has frequently been used with a wide range of pediatric populations (43–45). The German short form comprises 48 items (12 subscales with 4 items) which are rated on a 5-point Likert scale, with higher scores reflecting higher stress. The PSI parental domain consists of seven subscales (e.g., “Isolation” and “Health Problems”) and was labeled as “PSI parental stress” in this study. The PSI child domain—which is a measure of the stressor or demand—consists of five subscales (e.g., “Acceptability” and “Demandingness”), which was labeled as “PSI child-related stress.” In addition to these two main domains, norms exist for a PSI composite score.

### Predictors of Maternal Stress Outcome

#### Pervasive Developmental Disorder Behavior Inventory—Social Approach Behaviors

The Social Approach Behaviors dimension (SOCAPP) of the PDDBI (46) assesses a variety of primarily non-verbal social communication behaviors that are challenging for children with ASD. The scale comprises the following nine subdomains: visual social approach behaviors (e.g., “Pays attention to other's face when given instructions or when asked questions”), positive affect behaviors (e.g., “Smiles when praised”), responsiveness to social inhibition cues (e.g., “Completely stops inappropriate behavior for at least a day when warned or punished”), social play behaviors (e.g., “Selects his/her own toys to play with and allows other(s) to play along”), imaginative play behaviors [e.g., “Shows more complex imaginative toy play (e.g., ‘feeds doll, makes Superman™ toy fly')”], empathy behaviors (e.g., “Tries to physically or verbally comfort others when they are sad”), social interaction behaviors [e.g., “Seeks affection (e.g., hugs and kisses) from caregivers or siblings”], social imitative behaviors (e.g., “Can imitate tongue clicking”) and gestural approach behaviors [e.g., “Moves arm(s)/hand(s) in beckoning motion to signal others to come to him/her”]. The SOCAPP scale has an excellent reliability score (α = 0.91) and highly correlates with the Childhood Autism Rating Scale [(47); *r* = 0.60], the Vineland Adaptive Behavior Scales [(48); Socialization *r* = 0.58] and the Griffiths scales [(49); General IQ *r* = 0.63]. The SOCAPP composite score can be interpreted as strengths/weaknesses in the use of a large variety of social communication behaviors compared to an age-referenced norm sample of children with ASD, further labeled as “PDDBI SOCAPP.”

#### Aberrant Behavior Checklist

The Aberrant Behavior Checklist (50) is a 58-item behavior rating scale which is used to measure behavioral problems across five subdomains. All items are rated on a 4-point Likert scale (0–3), with higher scores indicating more severe problems. Following the recommendation of Kaat et al. (51), we report only scores of given subscales of the ABC with a proven robust factor structure, acceptable to excellent internal consistencies and also recently reported norm values for children with ASD <6 years and ID. Others have shown that the ABC is a sensitive instrument applicable in treatment and intervention studies (52, 53). We concentrated on external behavioral problems and the link to maternal stress outcome. Therefore, the scales Stereotypy (e.g., “repetitive hand movements”), Hyperactivity/Non-compliance (e.g., “excessively active”) and Irritability, including items encompassing intense emotional states, such as self-aggression, and aggressive behaviors (e.g., “cries and screams” and “willingly hurts him/herself”), were chosen to measure this construct. The internal consistencies for the chosen factors were: Stereotypic Behavior α = 0.87, Hyperactivity/Non-compliance α = 0.94 and Irritability α = 0.92 (51). We labeled these three scales as “ABC Stereotypy,” “ABC Hyperactivity,” and “ABC Irritability.”

#### Mullen Scales of Early Learning

Child non-verbal and verbal cognitive abilities were assessed using the Mullen Scales of Early Learning (54), which is a standardized developmental assessment for children 0–68 months of age. The enclosed and so named developmental domains are Visual Reception, Fine Motor Skills, Receptive Language and Expressive Language. Reliabilities were obtained for younger (0–2 years) and older (2–5 years) children with good stability coefficients for the younger group (0.82–0.85) and less reliable coefficients for the older group (0.71–0.79) with very high interrater reliability for both age groups (0.91–0.99). Since we encountered floor effects when calculating T-scores for our sample, we derived developmental quotients (DQ: age-equivalent/chronological age × 100) for each subscale in accordance with others [e.g., (55)]. As measures of non-verbal development, a composite score of the age-corrected MSEL subdomains Fine Motor Skills and Visual Recognition and, by analogy, as a measure of verbal DQ a composite of the Expressive and Receptive Language scales were derived and labeled as “MSEL Non-verbal” and “MSEL Verbal.”

#### Vineland Adaptive Behavior Scales 2nd Edition

The Vineland Behavior Scales Second Edition (56) is a questionnaire which measures daily living skills as reported by parents and includes personal skills (e.g., “eating, dressing, or personal hygiene”), domestic skills (e.g., “being careful using sharp objects”; “household tasks such as putting toys away”) and community skills (e.g., “remaining within safe distance of caregiver”). Reasons for selecting this specific subscale were time restrictions and the high relevance to children and families in everyday life (57). As for the MSEL subdomains, developmental quotients (age-equivalent/chronological age ^*^ 100) were derived for this subscale for better comparability with the reported MSEL subdomains and labeled as “VABS Daily Living.”

#### Controlling Predictor Variables

To control for potentially confounding variables, we included child's age (“Age Child”), multilingualism of family (“Multilingualism”) and maternal educational level (“Mother's Level of Education”) in our analyses.

### Intervention

#### Participants

Seventy-two children with ASD (63 male, 9 female) aged 29–60 months (*M* = 41.56; SD = 7.10) and their parents enrolled in this study. Most of the participating families were multilingual (72.2%) with 44.5% of the mothers holding at least a high school diploma and with an average of 1.10 (SD = 1.14) siblings living in the family. Participants were recruited consecutively from the community-based early-intervention program provided by our department, where they had been referred after diagnostic assessment between March 2014 and February 2020. Participants met DSM-5 criteria for ASD according to diagnostic assessments based on a multidisciplinary evaluation process using the ADOS-2 (58), which included a clinical assessment and a questionnaire battery (as described above). Intervention was offered to all families who met the following criteria: (a) child diagnosed with ASD, (b) non-verbal developmental age of at least 1 year, (c) parents requested early intervention, and (d) sufficient language skills (German or English). Assessments were administered in the family's primary language, with translation support when needed.

#### Implementation of the Intervention

The intervention team consisted of 12 therapists from various disciplines (speech-language therapy, occupational therapy, clinical psychology, special education) certified in the ESDM. The training and certification process lasted between 7 and 13 months (35). Prior to intervention, most of the parents took part in a parent-training workshop comprising six 2-h meetings per week which was developed and offered by the department's intervention team. Parents attended at least 1 weekly appointment with the interventionist over the 1-year intervention at which parent coaching was provided. During this period, therapy was delivered two to three times a week with a duration of 90 min. For further details on the intervention see (59).

### Design and Statistical Analysis

In this study we used a within-subject longitudinal design (T1-T2) to measure developmental change in child variables and parental stress that occurred during the 12-month intervention period. All variables of interest were measured using the standardized instruments listed above at T1. All measures—except the ADOS-2 assessment—were re-administered after the intervention period (T2), with an average of 11.49 (range: 9–13; SD = 0.87) months between T1 and T2. All data analyses were conducted with SPSS 26 statistical software. Paired sample tests were performed to separately analyze differences in child and maternal dimensions before and after treatment. To control for Type 1 errors due to multiple comparisons, the Bonferroni-Holm correction was applied to all child and maternal *p*-values. First, bivariate Pearson correlations between putative predictors were derived, and then between putative predictors and outcome variables. For all comparisons, Cohen's *d* was calculated and reported. All predictor variables were checked for multicollinearity, with very good variance inflation factors and tolerance values. Several multiple and hierarchical regressions were performed to examine the extent of variance explained by defined predictor variables.

## Results

At baseline the children showed severe delays in language development (*MSEL Verbal DQ* = 41.97; SD = 19.81) and less severe delays in non-verbal development (*MSEL Non-verbal DQ* = 63.98; SD = 16.24). Social Approach Behaviors (*PDDBI SOCAPP*) as rated by parents indicated average levels (*T* = 48.43, SD = 10.01) compared to a normed sample of children with ASD. The parent-rated *PSI child-related stress* score was above the chosen clinically relevant individual level of T ≥ 65 in accordance with others (60), with a group mean of *T* = 65.28 (SD = 6.38). The *PSI parental stress* score (non-child-related) was statistically increased (*T* = 57.09; SD = 10.32) using a one sample *t*-test [*t*_(63)_ = 5.50; *p* < 0.001] with a comparison value of *T* = 50. Table 1 reports the results for child development and maternal stress at intervention start (T1) and post-intervention (T2). There were no significant maternal stress changes for *PSI child-related stress* [T2-T1: *t*_(63)_ = 1.79; *p* = 0.14] or *PSI parental stress* [T2-T1: *t*_(63)_ = −0.44; *p* = 1.00] for the whole group. In an exploratory analysis, we split the mothers into two groups: clinically significant *PSI child-related stress* score at T1 (*T* ≥ 65) and subclinical (*T* < 65) score. Conducting a two-factor variance analysis (Group × Time) for the *PSI child-related stress* group (clinical, subclinical) and time (T1, T2) revealed an significant interaction effect [*F*_(1,62)_ = 12.86; *p* = 0.001]. The clinical *PSI child-related stress* group showed a significant reduction [*t*_(39)_ = 3.66; *p* = 0.001], the subclinical *PSI child-related* group showed a trend in the opposite direction [*t*_(23)_ = −1.71; *p* = 0.101].

**Table 1:** Developmental and behavioral differences between T1 and T2 (pooled *t*-tests).

***N* = 72**	**T1 (pre)**	**T2 (post)**	**Change scores**	***t*-test *p*-value**	**Effect size**
	***M* (SD)**	***M* (SD)**	***M* (SD)**	**(Bonferroni H. corrected)**	**(*Cohens d*)**
**Children**					
MSEL verbal (DQ)	41.97 (19.81)	48.67 (24.71)	6.70 (13.28)	<0.001^**^	0.30
MSEL non-verbal (DQ)	63.98 (16.24)	64.83 (20.90)	0.85 (14.67)	1	0.05
PDDBI SOCAPP (T)	48.43 (10.01)	53.46 (12.09)	5.03 (9.33)	<0.001^**^	0.45
VABS daily living (DQ)	63.50 (30.31)	69.64 (32.61)	6.14 (22.46)	0.164	0.20
ABC irritability (RS)	12.22 (7.98)	9.21 (8.24)	3.01 (6.65)	0.001^**^	−0.37
ABC stereotypy (RS)	4.10 (4.06)	2.96 (3.20)	1.14 (3.64)	0.069	−0.31
ABC hyperactivity (RS)	15.68 (9.71)	13.34 (9.87)	2.34 (8.85)	0.22	−0.24
**Mothers**					
PSI child-related stress (T)	65.28 (6.38)	63.58 (8.24)	−1.70 (7.93)	0.14	−0.23
PSI parental stress (T)	57.09 (10.32)	57.69 (11.99)	−0.60 (10.91)	1	0.05

** *p < 0.01*.

*DQ, Developmental Quotient; M, Mean; SD, Standard Deviation; RS, Raw Score; T, T-Score*.

Although *PSI parental stress* scores were significantly higher than in the norm group at both time points, these scores were not clinically relevant at the group level and were significantly lower than the *PSI child-related* stress scores pre- and post-intervention, which were clinically significant (*T* ~ 65) at the group level. Importantly, for the *PSI child-related stress* score, our sample exhibited ceiling effects (*T*-value of 70: at T1 *N* = 31 and at T2 *N* = 27). As also shown in Table 1, children made statistically significant improvements between T1 and T2 in the dimensions of language development (*MSEL Verbal* DQ; *p* < 0.001; *d* = 0.30), Social Approach Behaviors (*PDDBI SOCAPP*; *p* < 0.001, *d* = 0.45) and Irritability (*ABC-Irritability*; *p* = 0.001; *d* = 0.37). No significant changes in child *MSEL Non-verbal* DQ (*p* = 1.00; *d* = 0.05) or *Daily Living Skills* (*VABS Daily Living*; *p* = 0.164; *d* = 0.20) were found.

Intercorrelations between the putative predictors of maternal stress are presented in Supplementary Material. Correlations between putative predictors and maternal stress at T2 are reported in Table 2. *PSI child-related stress* at T2 correlated moderately with *PSI child-related stress at T1* (*r* = 0.44, *p* < 0.001) and with *PSI parental stress* at T1 (*r* = 0.38, *p* = 0.002) and weakly with *PDDBI SOCAPP* gains (*r* = −0.28, *p* = 0.018). *PSI child-related stress* at T2 did not correlate with *Mother's Level of Education* (*r* = 0.04) or *MSEL Non-verbal*/*Verbal* gains (both *r*'s = −0.16). We did not find a significant correlation with *VABS Daily Living* gains or reduction in behavioral problems. *PSI parental stress* at T2 correlated significantly only with *PSI parental* and *child-related stress* at T1.

**Table 2:** Correlations (pooled) between putative predictors and PSI child-related gains (difference) and PSI child-/parental outcome (after 12 months).

**Predictors**	**PSI-child-related stress at T2**	**PSI-parental stress at T2**
Age child	0.07	−0.16
Multilingualism	0.04	−0.05
Mother's level of education	0.04	0.12
PSI child-related stress at T1 (T)	0.44^**^	0.12^*^
PSI parental stress at T1 (T)	0.38^**^	0.53^**^
MSEL verbal gains (DQ)	−0.16	0
MSEL non-verbal gains (DQ)	−0.16	−0.20
PDDBI SOCAPP gains (T)	−0.28^*^	−0.01
VABS daily living gains (DQ)	−0.16	0.01
ABC irritability gains (RS)	0.19	0.18
ABC stereotypy gains (RS)	−0.06	0.09
ABC hyperactivity gains (RS)	0.19	0.16

* *p < 0.05*.

** *p < 0.01*.

*DQ, Developmental Quotient; T, T-score; RS, Raw Score*.

Four independent multiple regression analyses were performed to examine the predictive value of sociodemographic variables and verbal- and non-verbal gains on PSI stress outcome at T2 (see Table 3). *Age Child, Mother's Level of Education, Multilingualism* and *MSEL Verbal/Non-verbal* gains did not explain any substantial variance of *PSI child-related* or *PSI parental* outcome at T2. Due to their lack of significance, and in accordance with previous studies (17), none of the controlling predictor variables listed in Table 3 was included in further regression analyses.

**Table 3:** Multiple regression analyses using z-standardized (pooled) values of demographic variables and MSEL verbal/non-verbal DQ gains predicting PSI child-related and parental stress at T2.

	**PSI child-related stress at T2**			**PSI parental stress at T2**		
	***B* (β)**	**SE**	***p***	***B* (β)**	**SE**	***p***
**Demographics**	*R*^2^ = 0.01; *p* = 0.863			*R*^2^ = 0.04; *p* = 0.451		
Age child	0.09	0.12	0.490	−0.16	0.13	0.210
Mother's level of education	0.05	0.12	0.679	0.11	0.13	0.404
Multilingualism	0.06	0.12	0.633	−0.07	0.13	0.565
**MSEL (directly assessed)**	*R*^2^ = 0.04; *p* = 0.316			*R*^2^ = 0.06; *p* = 0.200		
MSEL verbal DQ gains	−0.11	0.15	0.478	0.15	0.17	0.376
MSEL non-verbal DQ gains	−0.11	0.14	0.452	−0.27	0.16	0.089

*DQ, Developmental Quotient*.

Two independent multiple hierarchical regression analyses were carried out to examine relations between the independent predictor variables and the dependent *PSI child-related* and *PSI parental stress* at T2 variables. In both models, the following variables were sequentially entered: *PSI child-related* and *PSI parental stress* at T1 in step 1, *PDDBI SOCAPP* gains in step 2, *VABS Daily Living* gains in step 3 and *ABC Irritability-, ABC Stereotypy-, and ABC Hyperactivity-* gains in step 4 (see Tables 4, 5).

**Table 4:** Hierarchical multiple regression analysis (pooled) predicting PSI child-related stress at T2.

**Predictor**	**PSI child-related stress at T2**											
	**Step 1**			**Step 2**			**Step 3**			**Step 4**		
	***B* (β)**	**SE**	***p***	***B* (β)**	**SE**	***p***	***B* (β)**	**SE**	***p***	***B* (β)**	**SE**	***p***
PSI child-related stress at T1	0.34	0.13	0.006^**^	0.38	0.12	0.001^**^	0.38	0.12	0.001^**^	0.45	0.11	<0.001^***^
PSI parental stress at T1	0.24	0.13	0.006^**^	0.25	0.12	0.038^*^	0.26	0.12	0.038^*^	0.26	0.12	0.027^*^
PPDBI SOCAPP gains				−0.36	0.11	0.001^**^	−0.37	0.12	0.002^**^	−0.39	0.11	0.001^**^
VABS daily living gains							0.03	0.12	0.800	0.06	0.12	0.594
ABC Irritability gains										0.30	0.11	0.009^**^
ABC Stereotypy gains										−0.10	0.11	0.400
ABC Hyperactivity gains										0.14	0.13	0.298
***R*** ^2^	0.24			0.36			0.36			0.49		
***F*** **change** (*p*-value)	9.60^***^ (*p* < 0.001)			11.46^**^ (*p* = 0.002)			0.06 (*p* = 0.840)			4.52^**^ (*p* = 0.007)		

* *p < 0.05*.

** *p < 0.01*.

*** *p < 0.001*.

**Table 5:** Hierarchical multiple regression analysis (pooled) predicting PSI parental related stress at T2.

**Predictor**	**PSI parental related stress at T2**											
	**Step 1**			**Step 2**			**Step 3**			**Step 4**		
	***B* (β)**	**SE**	***p***	***B* (β)**	**SE**	***p***	***B* (β)**	**SE**	***p***	***B* (β)**	**SE**	***p***
PSI child-related stress at T1	−0.14	0.11	0.223	−0.14	0.12	0.241	−0.14	0.12	0.234	−0.09	0.12	0.434
PSI parental stress at T1	0.60	0.12	<0.001^***^	0.60	0.12	<0.001^***^	0.62	0.12	<0.001^***^	0.64	0.12	<0.001^***^
PPDBI SOCAPP gains				−0.04	0.10	0.727	−0.10	0.12	0.423	−0.09	0.12	0.438
VABS daily living gains							0.13	0.12	0.306	0.20	0.12	0.110
ABC irritability gains										0.08	0.12	0.510
ABC stereotypy gains										0.03	0.12	0.820
ABC hyperactivity gains										0.21	0.14	0.127
***R*** ^2^	0.30			0.30			0.31			0.38		
***F*** **change** (*p*-value)	12.94^***^ (*p* < 0.001)			0.12 (*p* = 0.728)			1.05 (*p* = 0.310)			2.19 (*p* = 0.100)		

*** *p < 0.001*.

As shown in Table 4, *PSI child-related* and PSI *parental stress* at T1 scores predicted 24% of the variance of *PSI child-related stress* at T2 (*F*_change_ = 9.60, *p* < 0.001). Adding *PDDBI SOCAPP* gains in step 2 led to a highly significant improvement (plus 12%), which resulted in a total explanation of variance of 36% (*F*_change_ = 11.32 *p* < 0.001). *PDDBI SOCAPP* gains (β = −0.36, *p* = 0.001) were revealed to be a stronger predictor than *PSI parental stress* at T1 (β = 0.25, *p* = 0.038) and as predictive as the *PSI child-related stress* at T1 score (β = 0.38, *p* = 0.001). Adding *VABS Daily Living* gains in a third step showed no change in variance explanation. In a fourth step, we added three *ABC* subscale change scores (*ABC Irritability, ABC Stereotypy, ABC Hyperactivity*), which led to a significant improvement in variance explanation in this final model (plus 13%; *R*^2^ = 0.49; *F*_change_ = 4.52, *p* = 0.007). The three strongest predictors in this final model were *PSI child-related stress* at T1 (β = 0.45, *p* < 0.001), *PDDBI SOCAPP* gains (β = −0.39, *p* = 0.001) and *ABC Irritability* gains (β = 0.30, *p* = 0.009).

In contrast, as demonstrated in Table 5, only *PSI parental stress* at T1 contributed markedly to variance explanation (30%) of *PSI parental stress* at T2 in a first analytical step (*F*_change_ = 2.94, *p* < 0.001). Incorporating *PDDBI SOCAP*P gains in a second (*R*^2^ = 0.30) and *VABS Daily Living* gains in a third step (*R*^2^ = 0.31) resulted in no change in variance explanation of *PSI parental stress* at T2. The fourth step, entering the three *ABC* subscale change scores (*ABC Irritability, ABC Stereotypy, ABC Hyperactivity)*, led to a non-significant increase in variance explanation (plus 7%; *R*^2^ = 0.38; *F*_change_ = 2.19, *p* = 0.100). In this final model, the only significant predictor of *PSI parental stress* at T2 was—as in the other steps—*PSI parental stress* at T1 (β = 0.64, *p* < 0.001).

## Discussion

In this study we examined changes in maternal stress before and after ESDM intervention, and the relationship between stress outcomes and various dimensions of child development during the 1-year intervention period. As the ESDM focuses on the enhancement of child social communication behaviors, our particular interest was in the specific role of social communication development in predicting maternal stress, also considering gains in language, non-verbal cognition, adaptive skills and a reduction in aberrant behaviors. Our results did not support our first hypothesis of maternal stress decreasing during the intervention period. No significant differences in maternal stress between baseline and post-intervention were found, neither for child-related nor for parental stress in mothers. *That maternal stress does not change significantly in the course of primarily child-related interventions is supported by previous findings* (10, 11). Our data showed very high values comparable with those in other studies of young children with ASD (61). *Child-related maternal stress scores were clinically relevant and persisted throughout the intervention period*. Interestingly, in further exploratory analyses we found differences in child-related maternal stress development between mothers who reported clinically relevant child-related stress scores at baseline (*T* score ≥ 65) and mothers who scored below (subclinical group). *Mothers with very high child-related stress scores showed a significant reduction in child-related stress in contrast to the group of mothers with subclinical child-related stress*, who reported a slight increase in child-related stress. This within-group interaction effect might in part explain our findings of no groupwise change in child-related stress score during intervention. Closer investigation of this preliminary finding is needed in further studies.

Our analyses support our second hypothesis that improvements of social communication behaviors predict a significant proportion of variance in maternal child-related stress when stress scores pre-intervention, gains in (everyday life) adaptive skills and a reduction in behavioral problems are considered. *Improvements in social communication behaviors even turned out to be the strongest single predictor of maternal child-related stress*. Our results corroborate previous findings of associations between social communication skills of children with ASD and parental stress (20, 27).

Contrary to our expectation (hypothesis 3a), significant gains in verbal skills failed to show a significant relationship with maternal stress. Changes in non-verbal cognition and adaptive skills (*VABS Daily Living* scores with very high standard deviations) during the intervention period were neither statistically significant nor did they correlate with maternal stress outcomes, and hypotheses (3b) and (3c) were thus not confirmed. However, reduced irritability (unlike stereotypy and hyperactivity) showed a significant relationship with maternal stress and contributed significantly to explaining variance in our model which included maternal stress pre-intervention and development of social communication behaviors.

Even though language delays have been reported to be a major concern of parents of children with ASD and a primary reason for seeking support (19), *our data suggest that social communication behaviors* (including appropriate everyday use of language or non-verbal means of communication) *rather than verbal knowledge per se contributes to maternal stress*. While other studies showed child adaptive behavior levels to be significantly related to maternal stress (24, 62), we did not find that improvements in everyday living skills as measured by the VABS contributed to the prediction of maternal stress. Since an individual's reduced ability to take care of themself is expected to cause distress in a caregiver, deviations in our results might be due to the lack of significant changes within the intervention period, which might in turn be related to the measure's sensitivity to change.

In line with hypothesis (3d), a reduction in irritative challenging behavior was negatively related to maternal stress and was a highly significant predictor in our final model, increasing the extent of explained variance in addition to the effects of development of social communication behaviors. Specific correlations between child aberrant behaviors and parental stress have also been reported by other studies (10, 17).

We did not find any predictive values of child-related maternal stress outcome among the socio-demographic variables, which seems surprising. Possibly because our group of children was very young and age-homogeneous, we did not find a correlation between age and maternal stress; similar findings have also been reported by others (63). As mentioned above, our group of families showed great heterogeneity in their cultural, educational and language backgrounds, which is probably the reason for not finding any correlations between multilingualism or maternal education and maternal stress outcome.

Unlike for child-related maternal stress outcome, we did not find significant predictors of parent-related maternal stress outcome, except the intervention start value of this dimension. No family or child developmental scores were predictive of parental stress outcome at T2.

### Limitations

The findings of this study must be interpreted with caution, considering several limitations. First, the lack of a control group makes it impossible to determine if the reported changes reflect treatment effects. Other explanations (e.g., maturation) cannot be ruled out. Secondly, because of our clinic's standard procedure, families were seen by the same clinicians at follow up, which made blinding of the assessments impossible. Third, our parental rating scales were completed only by mothers. Although mothers are usually the primary caregivers, this makes it impossible to generalize our findings to fathers, who have also been reported as crucial to child development (64). Moreover, our maternal stress and social communication variables were based on maternal self-reported questionnaires. Fourth, there are concerns in relation to the PSI norms: As reported above, at both time points the PSI led to considerable ceiling effects, as reported by others (61, 65, 66), especially for the child domain. Concerns using the PSI-SF in studies with young children with ASD were also raised by Zaidman-Zait et al. (67). Fifth, one strength of this study—the use of a community sample—led to large heterogeneity in terms of family language and culture, with almost three quarters of the families being multilingual; this may have influenced the accuracy in answering the questionnaires, although assistance was offered whenever needed.

## Conclusion and Future Directions

Our findings indicate that improvements in social communication behaviors, for instance, improved social approach behaviors, showing positive affect behaviors, interconnecting in social plays, or imitating social cues, directly affect maternal child-related stress. This influence was ahead of all other measured dimensions of child development (e.g., challenging behaviors, adaptive skills or verbal skills as directly assessed by standardized instruments and non-verbal cognition). Parents who perceive a lack of, or stagnation in, social functioning in their children with ASD often experience a weakening or loss of “relationship/connection” with their child, which can lead to anxiety, confusion and worries in everyday situations and a loss of parental self-efficacy, including perceived competence and self-confidence. Our results underline the importance of addressing social communication in early intervention programs, such as the ESDM (35) or PACT (30), as changes in child social communication appear to be beneficial to maternal well-being.

Finally, the inclusion of valid parental rating measures of social functioning in everyday life situations, such as the PDDBI, and measures of parental stress during intervention appear to be important for two reasons: First, more longitudinal data is needed to explore the reciprocal mechanisms that link the enhancement of social behaviors in young children with ASD and maternal (parental) stress or mental health levels. Secondly, early intervention programs target primarily children's skills, but often neglect professional support or intervention procedures for parents, as also highlighted by others (68, 69). A better understanding of the interconnection between children's social development and parental well-being is needed to improve early intervention programs with more sophisticated procedures to meet the needs of parents of children with ASD.

## Data Availability Statement

The datasets generated and analyzed for this study are not publicly available due to the lack of confirmation by participating families to share their datasets with other institutions. Requests to access the datasets should be directed to dominik.laister@jku.at.

## Ethics Statement

The ethical approval for this evaluation was granted by the Ethics Committee of the Hospital of St. John of God in Linz in April 2014. All participating parents gave their written informed consent for participation in the study and publication of results.

## Author Contributions

DL and DH conceived and designed the study. DL performed the analysis and calculations and drafted the manuscript. DH and GV substantially revised the drafts and gave methodological feedback while planning the study. PM and JF supervised the project and gave input to the argumentation line of the study. All authors discussed the results and contributed to the final manuscript.

## Funding

Article processing charge was funded by the Johannes Kepler University Open Access Publishing Fund.

## Conflict of Interest

The authors declare that the research was conducted in the absence of any commercial or financial relationships that could be construed as a potential conflict of interest.

## Publisher's Note

All claims expressed in this article are solely those of the authors and do not necessarily represent those of their affiliated organizations, or those of the publisher, the editors and the reviewers. Any product that may be evaluated in this article, or claim that may be made by its manufacturer, is not guaranteed or endorsed by the publisher.
