# Supplementary material for: Enhancement of Social Communication Behaviors in Young Children With Autism Affects Maternal Stress
Source: Front Psychiatry. 2021 Dec 7;12:797148. doi: 10.3389/fpsyt.2021.797148 (PMC8688251; doi:10.3389/fpsyt.2021.797148)
Supplement: Supplementary file 1 [file Table_1.DOCX]

Table SUPPLEMENT. Intercorrelations (pooled) of all predictor variables

| ***Predictors*** | 1 | 2 | 3 | 4 | 5 | 6 | 7 | 8 | 9 | 10 | 11 | 12 |
| --- | --- | --- | --- | --- | --- | --- | --- | --- | --- | --- | --- | --- |
| 1. Age Child | 1 | -.17 | -.08 | -.02 | -.13* | -.21 | .07 | -.12 | -.19 | -.11 | -.03 | -.22 |
| 2. Multilingualism | -.17 | 1 | -.02 | .08 | -.08 | -.17 | -.18 | .02 | -.02 | .03 | -.19 | .12 |
| 3. Mother’s Level of Education | -.08 | -.02 | 1 | .12* | .14** | .17 | -.03 | .04 | .03 | -.07 | -.08 | .04 |
| 4. PSI child-related stress at T1 (T) | -.02 | .08 | .12* | 1 | .45** | -.01 | -.06 | .10* | .00 | -.20** | -.18** | -.17** |
| 5. PSI parental stress at T1 (T) | -.13* | -.08 | .14** | .45** | 1 | .00 | -.27** | .09 | -.11* | -.04 | -.09 | -.14** |
| 6. MSEL Verbal gains (DQ) | -.21 | -.17 | .17 | -.01 | .00 | 1 | .51** | .44** | .40** | -.11 | -.11 | -.12 |
| 7. MSEL Nonverbal gains (DQ) | .07 | -.18 | -.03 | -.06 | -.27** | .51** | 1 | .26* | .29* |  | -.10 | -.18 |
| 8. PDDBI SOCAPP gains (T) | -.12 | .02 | .04 | .10* | .09 | .44** | .26* | 1 | .45** | .10 | -.03 | -.23* |
| 9. VABS Daily Living gains (DQ) | -.19 | -.02 | .03 | .00 | -.11* | .40** | .29* | .45** | 1 | .05 | .13 | .31** |
| 10. ABC Irritability gains (RS) | -.11 | .03 | -.07 | -.20** | -.04 | -.11 | -.03 | .10 | .05 | 1 | .21** | .44** |
| 11. ABC Stereotypy gains (RS) | -.03 | -.19 | -.08 | -.18** | -.09 | -.11 | -.10 | -.03 | -.13 | .21** | 1 | .51** |
| 12. ABC Hyperactivity gains (RS) | -.22 | .12 | .04 | -.17** | -.14** | -.12 | -.18 | -.23* | -.31** | .44** | .51** | 1 |

Notes: *… p<.05; **…p<.01; DQ…developmental quotient; T…T-Score; RS…raw score

MSEL…Mullen Scales of Early Learning;
PDDBI SOCAPP…Pervasive Developmental Disorder Behavior Inventory Social Approach Behavior;
VABS… Vineland Behavior Scales 2^nd^ Edition;
ABC…Aberrant Behavior Checklist;
PSI...Parental Stress Index - Short Form

Significant moderate correlations (r≥.50) were found for *MSEL Verbal DQ/MSEL Nonverbal DQ* (r=.51; p<.001) and *ABC Hyperactivity/ABC Stereotypy* (r=.51; p<.001).
